# Solitary metastatic gallbladder malignant melanoma originated from the nasal cavity: A case report^☆^

**DOI:** 10.1016/j.ijscr.2013.08.005

**Published:** 2013-08-20

**Authors:** Katsuyoshi Furumoto, Yuya Miyauchi, Daisuke Ito, Toshiyuki Kitai, Masafumi Kogire

**Affiliations:** Department of Surgery, Kishiwada City Hospital, 1001 Gakuhara-cho, Kishiwada-shi, Osaka 596-8501, Japan

**Keywords:** Malignant melanoma, Gallbladder, Metastatic melanoma, Nasal melanoma, Dedifferentiation

## Abstract

**INTRODUCTION:**

Solitary gallbladder metastasis of malignant melanoma is rare and generally originates from skin melanoma. We report a case of gallbladder metastasis from a malignant melanoma of the nasal mucosa that was surgically treated.

**PRESENTATION OF CASE:**

A 77-year-old Japanese woman diagnosed with malignant melanoma of the left sinonasal cavity three years ago underwent follow-up PET–CT and FDG uptake was detected only at the gallbladder. The nasal melanoma had been stable for the last 1.5 years after chemoradiation and her general condition was good. Cholecystectomy was performed with partial liver resection. Lymphadenectomy of the hepatoduodenal ligament was also performed. The tumor was soft and whitish, and was microscopically diagnosed as a poorly differentiated malignant melanoma that was not similar to the nasal cavity melanoma. No further metastasis is observed for more than 13 months after surgery.

**DISCUSSION:**

In the literature, cutaneous melanoma is described as the origin of most metastatic gallbladder melanomas; however, no skin lesion was evident in this case. We believe that the poorly differentiated compartment of the nasal melanoma had metastasized to the gallbladder.

**CONCLUSION:**

For patients with melanomas and gallbladder tumors, the possibility that metastasis could occur should be considered when selecting optimal treatment. Even when original melanoma is present, surgical treatment for gallbladder metastasis may be useful depending on the patient's conditions.

## 1 Introduction

Malignant melanomas can metastasize to any organ; the most common sites of distant metastases are the lung (70–87%), liver (54–77%), and skin (50–75%).^1^ Within the gastrointestinal tract (26–58%), melanomas metastasize most commonly to the small intestine (approximately 67%) and rarely to the gallbladder.^2^ Among metastatic gallbladder tumors, melanomas are the most common origin of malignancy, accounting for 50–60% of all reported gallbladder metastases.^3,4^

Metastatic gallbladder melanoma has been reported several times,^5–9^ including one by Dong et al.,^5^ with 19 patients and one by Katz et al.,^6^ with 13 patients; majority of the primary lesions were skin melanomas.

Only one out of 23 gallbladder tumors was previously detected using abdominal ultrasound in patients with melanoma, and it originated from the nasal mucosa.^7^ We report a case of a female with an isolated gallbladder metastasis that originated from a nasal cavity malignant melanoma. To our knowledge, this is the first report that describes such a case in detail.

## 2 Case presentation

**Fig. 1:**
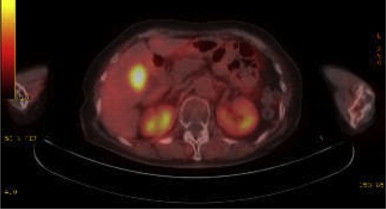
A follow-up PET–CT revealed the uptake of FDG only at the gallbladder (SUV_max_ 7.1).

**Fig. 2:**
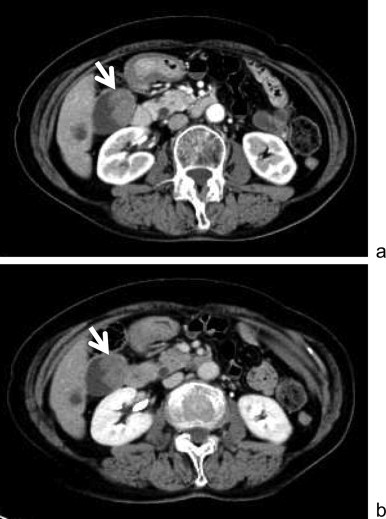
Contrast abdominal CT showed an early enhanced mass in the gallbladder (arrow) and the enhancement persisted on the equilibrium phase, suggesting gallbladder cancer. (a) Early phase. (b) Equilibrium phase.

**Fig. 3:**
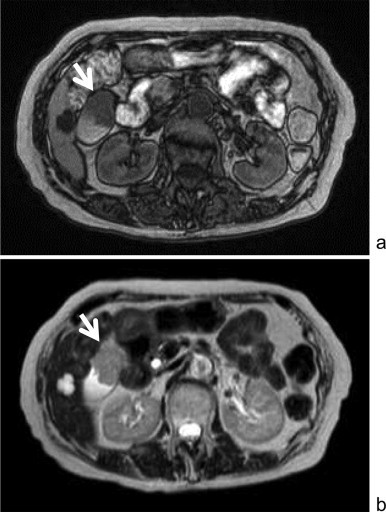
MRI demonstrated that the gallbladder tumor (arrow) showed low signal intensity on T1-weighted images and slightly high on T2-weighted images. (a) T1-weighted images. (b) T2-weighted images.

A 77-year-old Japanese woman was diagnosed with malignant melanoma in the right sinonasal cavity using biopsy approximately three years ago. She complained of nasal bleeding at that time. She underwent proton beam radiation therapy with sequential chemotherapy; however, she experienced double vision, and 1.5 years later, MRI revealed a local recurrence. After several courses of additional chemotherapy, the disease remained stable for the last 1.5 years. She underwent follow-up PET–CT and FDG uptake was detected at the gallbladder (Fig. 1). Hematological examination revealed no specific findings. Contrast abdominal CT revealed an early enhanced mass in the gallbladder, and the enhancement persisted in the equilibrium phase (Fig. 2). On MRI, the tumor showed low signal intensity on T1-weighted images and slightly high intensity on T2-weighted images (Fig. 3). Endoscopic ultrasonography (EUS) showed no apparent tumor infiltration to the liver.

**Fig. 4:**
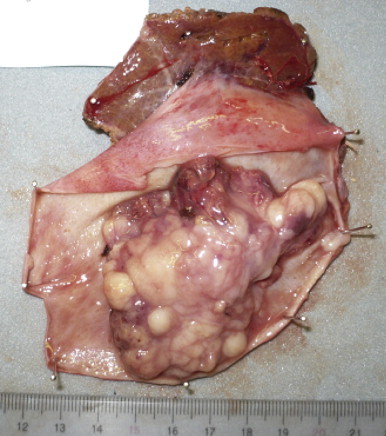
The gallbladder tumor was macroscopically soft and whitish measured 7.5 cm × 5 cm in size.

**Fig. 5:**
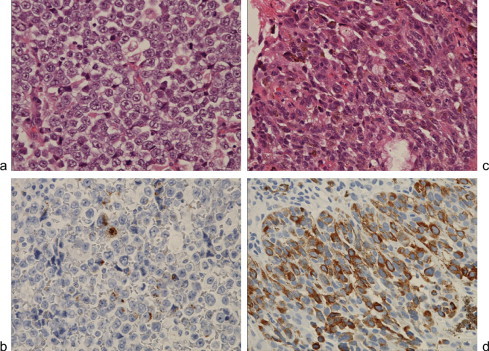
Immunohistochemical examination revealed that the gallbladder tumor was not similar to the original nasal melanoma. HMB-45 staining was far less positive in the gallbladder tumor than in the nasal tumor. (a) Gallbladder tumor, HE staining. (b) Gallbladder tumor, HMB-45 staining. (c) Nasal tumor, HE staining. (d) Nasal tumor, HMB-45 staining (200×).

The patient did not present with any symptoms. Because of the remaining nasal melanoma, surgical indication for the gallbladder tumor was controversial. We, however, recommended surgical treatment and she agreed for fear of its malignant potential. The primary preoperative diagnosis of the lesion was gallbladder cancer, and the secondary assessment was metastatic melanoma. Her general condition was good. After obtaining informed consent, which included advisement of the risk of relapse of nasal melanoma, we performed laparotomy and cholecystectomy with partial liver resection. In addition, lymphadenectomy of the hepatoduodenal ligament was performed. No ascites or macroscopic peritoneal dissemination was observed, and the tumor was localized in the gallbladder. The distal margin of the cystic duct was cancer free and the common bile duct was preserved. The tumor was soft, whitish, and measured 7.5 cm × 5 cm (Fig. 4). Microscopically, it was diagnosed as a poorly differentiated malignant melanoma. Immunohistochemical examination revealed that the tumor was not similar to nasal melanoma, i.e., HMB-45 staining was far less positive in the gallbladder tumor than in the nasal tumor (Fig. 5). The tumor cells reached the subserosal layer with mild vascular involvement, and the lymph nodes gave negative results for metastasis. No gallstones were observed. The postoperative course was uneventful. No distal recurrence has been detected for more than 13 months after surgery.

## 3 Discussion

Metastatic gallbladder malignancies are rare, with a frequency of 5.8% in an analysis of 1000 carcinoma autopsies.^10^ Malignant melanoma can metastasize to any organ, and the most common sites of distant metastases are the lung, liver, and skin.^1^ Within the gastrointestinal tract, malignant melanomas metastasize most commonly to the small intestine, while rarely to the gallbladder.^2^

In contrast, among metastatic gallbladder tumors, melanoma is the most common origin of malignancy.^3,4^ At autopsy, the incidence of gallbladder metastases is 15–20% in patients who died of malignant melanoma.^11^ Among patients with malignant melanomas, 5.2–8% of patients were diagnosed with metastatic gallbladder lesions using abdominal ultrasound examination.^7,12^

Metastatic gallbladder melanomas have been previously studied, which includes a study by Dong et al.,^5^ with 19 patients and one by Katz et al.,^6^ with 13 patients; majority of the primary lesions were cutaneous melanomas. Mucosal melanomas of the sinonasal tract are infrequent and account for less than 1% of all melanomas and less than 4% of all sinonasal malignancies.^13^ Incidence of malignant melanomas in Japan is low compared with that in Western countries. However, head and neck mucosal melanoma is relatively common and comprises 6% of all melanomas.^14^ The present case involved an isolated metastasis from a nasal cavity malignant melanoma to the gallbladder. To our knowledge, this is the first report describing such a case in detail.

In Japan, Shimada et al. reported the occurrence of malignant melanomas in digestive organs.^15^ Among 51 metastatic gallbladder tumors, malignant melanoma (11 cases) was the second leading origin, which is different from Western reports.^3,4^ Resection of these 11 gallbladder melanonas revealed the origin site of the melanomas to be the skin (eight cases), anorectum (two cases) and esophagus (one case). Till date, gallbladder metastasis from a nasal melanoma has not been reported in Japan.

It is difficult to state whether a gallbladder tumor is a primary or metastatic malignancy. Our main preoperative diagnosis was gallbladder cancer. We performed extended cholecystectomy with lymphadenectomy of the hepatoduodenal ligament, which is the standard method for T2 or more advanced gallbladder tumors. Abdominal imaging revealed atypical findings for a melanoma. In particular, MRI revealed low signal intensity on T1-weighted images. The expected signal pattern of a melanoma is high intensity on T1-weighted images as a result of melanin, and can vary depending on the percentage of melanin-containing cells.^16^ In our case, the gallbladder tumor microscopically revealed little melanin, and we speculate that the atypical low signal intensity on T1-weighted images was related to the melanin content of the tumor.

Because the sinonasal melanoma was advanced, we considered the possibility of metastatic melanoma in the gallbladder before the surgery. The tumor occupied the neck of the gallbladder on abdominal imaging, which could present a risk of cholecystitis due to cystic duct obstruction. In addition, the gallbladder lesion was solitary, which is rare in metastasis of melanomas. Gallbladder lesions often occur as part of widespread metastatic disease.^8^ Moreover, the nasal melanoma was stable for approximately 18 months. On the basis of these conditions, we decided to perform cholecystectomy after obtaining informed consent.

The optimal therapy for metastatic gallbladder melanoma is not definitive; however, surgical treatment, including cholecystectomy, can improve quality of life and survival even in patients with other metastatic disease.^5,9^

There have been some reports of laparoscopic therapy for metastatic gallbladder melanoma, and some have described port-site recurrence.^5^ The Japanese guideline for gallbladder tumors does not recommend laparoscopic cholecystectomy in cases of suspected malignancy.^17^ Because the gallbladder tumor in our case did not appear to be benign and early, we did not perform laparoscopic surgery.

The patient is alive for more than 13 months without new metastases, although the primary nasal lesion remains. In the report concerning 13 patients with cutaneous melanomas with gallbladder metastases, the median survival after cholecystectomy was 12 months, and operative treatment significantly improved their survival.^6^

In the present case, the metastatic gallbladder lesion was less differentiated (almost undifferentiated) than the original sinonasal melanoma. Because of the presence of nasal melanoma and rarity of primary gallbladder melanoma, we ruled out primary gallbladder melanoma. Brawn et al. showed that compared with the primary prostate cancer, dedifferentiation occurred within metastases, suggesting that this phenomenon may be important for widespread dissemination.^18^ We speculated that the gallbladder tumor in our case may have resulted from dedifferentiation of a sinonasal melanoma and become more aggressive; thus, we believed that the tumor required resection before it could spread further around the gallbladder.

## 4 Conclusion

We reported a case of a female with an isolated metastasis from a nasal cavity malignant melanoma to the gallbladder. She underwent extended cholecystectomy with lymphadenectomy of the hepatic hilus and remains alive for more than one year after the surgery. For patients with melanomas and gallbladder tumors, metastasis should be considered as a possibility when selecting optimal treatment. Even when original melanoma is present, surgical treatment for gallbladder metastasis may be useful depending on the patient's conditions.

## Conflict of interest

None.

## Funding

None.

## Ethical approval

Written consent was obtained from the patient regarding publication.

## Author contributions

KF has organized this work and written the manuscript. All authors reviewed the manuscript and approved the final version.
